# Investigation of the Hydration and Solidification Effect of Peanut Ash Cement-Based Stabilizer in Soft Clay Treatment

**DOI:** 10.3390/ma19020318

**Published:** 2026-01-13

**Authors:** Yongqin Qiu, Qichang Fan, Kun Zhang

**Affiliations:** 1 School of Civil Engineering, Sun Yat-sen University, Guangzhou 510275, China; 2 State Key Laboratory of Subtropical Building and Urban Science, South China University of Technology, Guangzhou 510640, China

**Keywords:** cement-based stabilizer, hydration, peanut ash, soft clay consolidation

## Abstract

To promote the sustainable utilization of agricultural solid waste, this study proposes a novel approach for reinforcing soft clay using a peanut ash (PA)–cement composite stabilizer. The unconfined compressive strength (UCS) of pure cement and PA–cement composite systems was tested at curing ages of 3, 7, and 28 days, while the durability of the stabilized clay was evaluated through dry–wet cycling. Given that PA is rich in pozzolanic components, its addition may influence the hydration process of cement. Therefore, hydration heat analysis was conducted to examine the early hydration behavior, and XRD and TG analyses were employed to identify the composition and quantity of hydration products. SEM observations were further used to characterize the microstructural evolution of the stabilized matrix. By integrating mechanical and microstructural analyses, the solidification mechanism of the PA–cement stabilizer was elucidated. Mechanical test results indicate that the reinforcing effect increases with the stabilizer dosage. Pure cement exhibited superior strength at 3 days; however, after 7 days, specimens incorporating 5% PA showed higher strength than those stabilized solely with cement. At 28 days, the UCS of the 15% cement + 5% PA specimen reached 3.12 MPa, 11.03% higher than that of the 20% cement specimen and comparable to the 25% cement specimen (3.15 MPa). After five dry–wet cycles, the strength reduction of the 15% cement + 5% PA specimen was 22.76%, compared to 31.31% for the 20% cement specimen, indicating improved durability. Microscopic analyses reveal that PA reduces hydration heat and does not participate in early hydration, leading to lower early strength. However, its pozzolanic reactivity contributes to secondary hydration at later stages, promoting the formation of additional C-S-H gel and ettringite. These hydration products fill the inter-lamellar pores of the clay and increase matrix density. Conversely, excessive PA content (≥10%) exerts a dilution effect, reducing the amount of hydration products and weakening the mechanical performance. Overall, the use of an appropriate PA dosage in combination with cement enhances both strength and durability while reducing cement consumption, providing an effective pathway for the high-value utilization of agricultural solid waste resources.

## 1. Introduction

With the rapid advancement of urbanization, urban spaces are continuously expanding into previously undeveloped areas. Soft clay strata in coastal zones, abandoned riverbeds, and similar regions have become key targets for urban development due to their substantial economic value and development potential. However, soft clay exhibits poor engineering properties, characterized by low bearing capacity, high water content, and pronounced deformation and settlement under loading. Consequently, the stabilization of soft clay has become a critical issue in urban development and construction [1,2].

In recent years, a lot of research has been carried out on the subject of soft clay reinforcement. Common soft clay reinforcement methods include the drainage method, grouting method, reinforcement method, chemical reinforcement method, etc. Among them, the chemical reinforcement method is the most widely used in engineering practice because of its simple operation and remarkable reinforcement effect [3,4]. Organic or inorganic curing agents are often used in chemical reinforcement methods. Organic curing agents are mostly liquid and are mainly prepared from resins, polymers, and polymer materials. Yang et al. used carboxymethyl cellulose and polyacrylamide as reinforcing agents to improve the soft clay, and the mechanical test results showed that the permeability resistance and shear strength of the reinforced soft clay were significantly improved [5]. Liu et al. used sulfur-free lignin to improve soft clay and found that it can effectively reduce the porosity of soft clay and improve the strength of reinforced soft clay [6]. However, it is easy for liquid additives to expire, and they have high storage requirements, which limit their application in engineering.

Inorganic curing agents used for soft clay stabilization are predominantly cement; however, cement-stabilized soft clay exhibits several shortcomings. On one hand, cement production is associated with high carbon emissions; on the other, the presence of organic matter in silty soft clay inhibits cement hydration, resulting in low strength development in the stabilized soil [7]. To address these limitations, researchers have proposed the use of supplementary cementitious materials (SCMs) in combination with cement to enhance the strength and durability of soft clay. Asavapisit et al. [8] reported that the addition of silica fume significantly improved the unconfined compressive strength (UCS) of cement-stabilized soft clay. Similarly, Basha et al. [9] demonstrated that the incorporation of rice husk ash enhanced the UCS of soft clay with increasing ash content. James et al. [10] used bagasse ash with cement and found that it improved compressive strength, albeit with a slight increase in water absorption. Abdullah et al. [11] utilized fly ash-based polymer binders to replace cement and lime, achieving strength values substantially higher than those obtained with pure cement stabilization. These studies collectively indicate that the use of SCMs in conjunction with cement is an effective strategy for soft clay reinforcement. Significant progress has been achieved using common SCMs such as silica fume, fly ash, rice husk ash, and plant ash [12,13,14]. Peanut is an important agricultural product [15], and its byproduct, peanut shell, is often discarded or incinerated after harvest. Recent studies have explored the partial replacement of cement with peanut ash in concrete production, revealing that peanut ash can promote cement hydration, enhance matrix density, and improve mechanical performance [16,17,18]. This suggests that peanut ash possesses promising potential as a supplementary cementitious material. In addition, cement undergoes a high-temperature calcination process at 1000–1200 °C during production. In contrast, the preparation of peanut shell ash only requires temperatures of 500–600 °C, significantly reducing energy consumption and carbon emissions during calcination [19,20,21]. Moreover, peanut shells are derived from agricultural waste, resulting in low raw material costs and offering economic and environmental benefits. The research reported that using PA instead of 2% cement to prepare cement mortar will reduce the carbon emissions of mortar by 2–6% [22]. From this, the authors presumed that using PA instead of part of cement to reinforce the strength of soil would have better carbon emission benefits. It can be seen that compared to cement, peanut shell ash exhibits superior economic and ecological advantages. However, research on the combined use of peanut ash and cement for soft clay stabilization remains scarce and warrants further investigation.

Soft clay is characterized by high water content and pronounced sensitivity to environmental changes; therefore, the durability of the stabilized soil under external disturbances is of critical importance. Tang et al. [23] examined variations in the hydraulic conductivity and mechanical behavior of soft clay under freeze–thaw cycles and analyzed corresponding microstructural changes via MIP and SEM. Niu et al. [24] investigated the strength evolution of stabilized soft clay under repeated dry–wet cycles, while other scholars have assessed its resistance to sulfate and chloride ion attack [25,26,27]. These studies indicate that environmental conditions significantly influence the performance of stabilized soft clay, and that its strength gradually deteriorates under prolonged exposure to such conditions. Hence, the post-stabilization durability and long-term serviceability of soft clay require further attention.

In this context, the present study proposes the use of peanut ash in conjunction with cement as a sustainable stabilizer for soft clay. The feasibility of PA–cement synergy was evaluated by analyzing the UCS of reinforced soft clay at different mix ratios and after multiple dry–wet cycles. Furthermore, hydration heat analysis, XRD, thermogravimetric (TG) analysis, and SEM observations were conducted to elucidate the influence of peanut ash on the cement hydration process and to reveal the underlying mechanism of its synergistic effect in soft clay stabilization.

## 2. Materials and Methods

### 2.1. Raw Material and Sample Preparation

**Figure 1:**
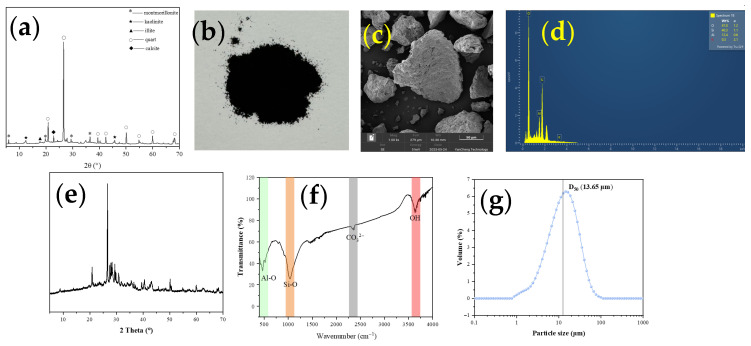
The XRD of soft clay: (**a**) XRD. The ingredient characteristics of peanut ash: (**b**) morphology; (**c**) SEM; (**d**) EDS; (**e**) XRD; (**f**) FTIR; (**g**) particle size distribution.

The soft clay samples used in this study were collected from a deep soft clay layer at a water purification plant construction site in Taizhou City, Zhejiang Province, China, using a drill to extract the deep soft soil from the site to a depth of 30 m. The X-ray diffraction (XRD) results (Figure 1a) indicate that the primary mineral components of the clay are quartz, montmorillonite, kaolinite, and illite. Notably, the relative intensity of the montmorillonite peak is significantly higher than that of kaolinite and illite, suggesting a high montmorillonite content in the soft clay from this region. Ordinary Portland cement (PO 42.5) and peanut ash (PA) were used as stabilizing agents. The preparation of PA followed the procedure described in the literature [18]. The PA was heated to 600 °C at a rate of 5 °C/min, kept warm for 2 h, and then ground at a rate of 300 rpm/min for 15 min. Its morphology is shown in Figure 1b,c. Energy-dispersive X-ray spectroscopy (EDS), XRD, and Fourier-transform infrared spectroscopy (FTIR) were conducted to characterize the composition and structure of PA, as presented in Figure 1d–f. EDS analysis revealed that the main elemental constituents of PA are oxygen, silicon, and aluminum. The XRD pattern displayed distinct SiO_2_ crystal diffraction peaks, while the FTIR spectrum further confirmed the presence of functional groups such as Al-O, Si-O, and CO_3_^2−^. The size information of PA can be seen in Figure 1g. The detailed chemical compositions of cement and PA are listed in Table 1.

**Figure 2:**
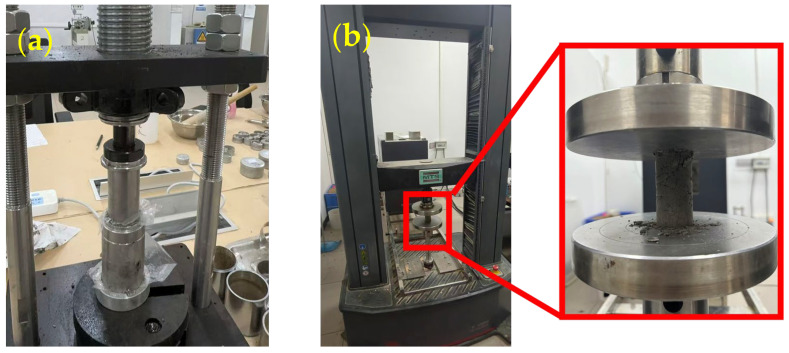
The preparation and strength test of soft clay samples: (**a**) sample preparation; (**b**) strength test.

The soil exhibits the most stable properties at the optimum water content. Adding the stabilizing agent in this condition results in the best stabilization performance, which is conducive to comparing the stabilization effects of different mix proportions [24]. Physical property tests of the soft clay were conducted in accordance with the Standard for Geotechnical Testing Method (GB/T 50123-2019) [28]. According to GB/T 50123-2019, the optimum water content of a soil is determined by a compaction test. The soil sample is air-dried and sieved, and five groups of specimens with different water contents are prepared and mixed uniformly. The specimens are then compacted in layers and weighed, and their water contents measured. Following that, their dry density and the dry density–water content curve were calculated, and the water content corresponding to the peak of the curve was taken as the optimum water content. The optimum moisture content of the clay was determined to be 40%, which was adopted as the reference moisture content for sample preparation. The preparation process of the stabilized soft clay specimens was as follows: undisturbed soft clay samples were first oven-dried, crushed, and sieved through a 2 mm standard sieve. The required amounts of soft clay and stabilizer were then weighed and mixed thoroughly with deionized water. The homogeneous mixture was poured into a cylindrical mold with an inner diameter of 39 mm and a height of 80 mm, followed by compaction using a hydraulic jack (Figure 2a). After demolding, the specimens were immediately wrapped with plastic film to prevent moisture loss. All samples were prepared with a consistent dry density, and the total dry mass (soft clay + stabilizer) was kept constant. Two reinforcement systems were investigated in this study: pure cement (denoted as “C”) and PA–cement composite curing (denoted as “A”). For each sample group, three parallel samples were prepared and the average was taken as the test result.

### 2.2. Test Method

After the samples were cured to the curing age (3 d, 7 d, 28 d), the unconfined compressive strength was measured using an MTS universal mechanical testing machine (Eden Prairie, MN, USA). The test program adopted a displacement control and the loading rate was 1 mm/min. The schematic diagram of mechanical testing is shown in Figure 2b.

The TAM AIR instrument was used to collect the hydration heat of consolidated soft clay, and Bruke D 8 was used to scan samples at a rate of 5°/min to analyze the phase composition of consolidated soft clay. TG209F1 Libra was used to collect the mass change of consolidated soft clay during calcination at 30–900 °C, and the Tescan field emission electron microscope was used to observe the filling effect of hydration products on the pore structure of consolidated soft clay.

## 3. Results and Discussion

### 3.1. Unconfined Compressive Strength

Unconfined compressive strength (UCS) tests were conducted to evaluate the effectiveness of stabilizer materials in improving the strength of soft clay. The influence of stabilizer dosage (15%, 20%, 25%, and 30%) on the strength development of the reinforced clay after curing for 3, 7, and 28 days was investigated. A PA–cement synergistic stabilization system was adopted in this study. Taking a total stabilizer content of 15% as an example, “0A” denotes a specimen containing 15% cement and 0% PA, “5A” means containing 5% PA + 10% cement, “10A” denotes containing 10% PA + 5% cement, and “15A” means containing 15% PA + 0% cement. As for a total stabilizer content of 20%, “0A” denotes a specimen containing 20% cement and 0% PA, “5A” means containing 5% PA + 15% cement, “10A” denotes containing 10% PA + 10% cement, and “15A” means containing 15% PA + 5% cement. Regarding a content of 25%, “0A” denotes a specimen containing 25% cement and 0% PA, “5A” means containing 5% PA + 20% cement, “10A” denotes containing 10% PA + 15% cement, and “15A” means containing 15% PA + 10% cement.

**Figure 3:**
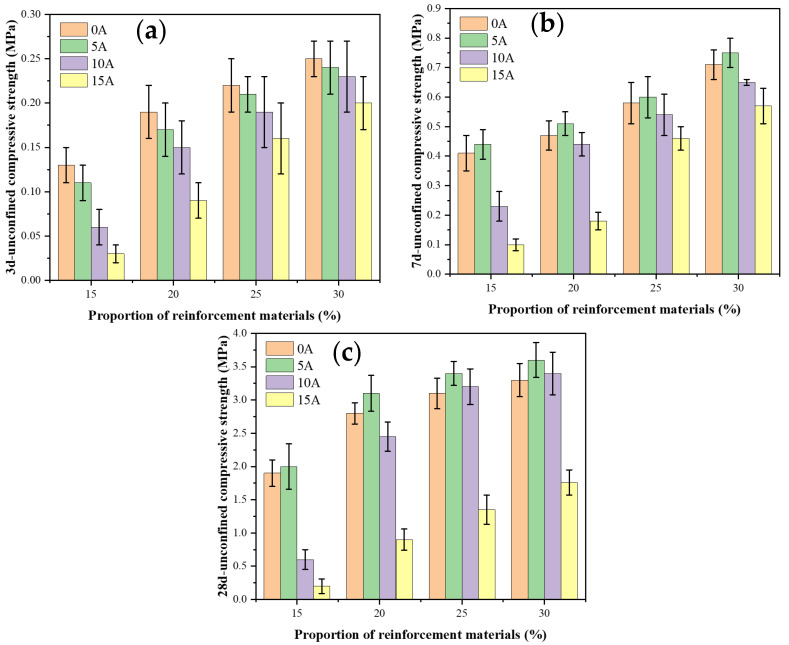
The UCS of soft clay at different ages: (**a**) 3 days; (**b**) 7 days; (**c**) 28 days.

The UCS results at 3 days are presented in Figure 3a. As the total stabilizer content increases, the compressive strength of the stabilized soft clay increases correspondingly. At this early age, the strength of specimens stabilized with pure cement is higher than that of the PA–cement specimens. Moreover, the early strength decreases progressively with increasing PA content, and a pronounced reduction is observed when the PA content reaches 15%. This phenomenon may be attributed to the fact that cement, primarily composed of calcium oxide (CaO), rapidly reacts with pore water to form early hydration products Ca(OH)_2_ that enhance the initial strength of the stabilized clay. In contrast, PA mainly contains SiO_2_ and Al_2_O_3_, which exhibit low reactivity at this stage and thus fail to contribute effectively to early strength development.

The UCS results at 7 days are shown in Figure 3b. For pure cement-stabilized specimens, the strengths corresponding to cement contents of 15%, 20%, 25%, and 30% are 0.41, 0.47, 0.58, and 0.71 MPa, respectively. When 5% PA is incorporated, the strengths of the 10C + 5A, 15C + 5A, 20C + 5A, and 25C + 5A specimens increase to 0.44, 0.51, 0.60, and 0.75 MPa, respectively. Compared with specimens stabilized with the same total amount of pure cement, the samples containing 5% PA exhibit higher strength, indicating that the “PA–cement” system demonstrates a synergistic effect at this curing age. However, when the PA content is increased to 10%, the strength becomes slightly lower than that of the corresponding pure cement specimens. A further increase in PA content to 15% results in a significant strength reduction. The 7-day strength results suggest that PA functions effectively only as a supplementary cementitious material and achieves the best reinforcement performance when incorporated at 5% in combination with cement at the early curing age. However, the optimal dosage ratio of peanut ash and cement needs to comprehensively consider multiple indicators such as early strength, late strength, and durability.

At a curing age of 28 days, the strength of the reinforced soft clay is shown in Figure 3c. The results indicate that the UCS of samples stabilized solely with cement at dosages of 15%, 20%, 25%, and 30% increased to 1.94 MPa, 2.81 MPa, 3.15 MPa, and 3.31 MPa, respectively. Clearly, increasing the cement content significantly enhances the strength of the stabilized clay. However, excessive cement usage results in higher costs and increased carbon emissions; therefore, cement dosage cannot be increased indefinitely to achieve optimal reinforcement. When the cement content is 20%, the UCS is only slightly lower than that of samples with 25% and 30% cement, suggesting that 20% cement represents a more suitable dosage in terms of strength and sustainability.

For samples incorporating 5% PA, the UCS values for mixtures of 10C + 5A, 15C + 5A, 20C + 5A, and 25C + 5A were 2.03 MPa, 3.12 MPa, 3.41 MPa, and 3.64 MPa, respectively. The strength of the 15C + 5A mixture exceeded that of the pure-cement sample containing 20% cement and approached that of the 25% cement sample. This suggests that incorporating 5% PA alongside cement can enhance the strength of soft clay while reducing cement consumption. The improvement may be attributed to the pozzolanic reaction between Ca(OH)_2_ produced during cement hydration and the SiO_2_ and Al_2_O_3_ supplied by PA, which promotes secondary hydration and the formation of additional C-S-H and ettringite within the clay matrix [29].

When the PA content increased to 10%, the UCS values of 5C + 10A, 10C + 10A, 15C + 10A, and 20C + 10A samples were 0.62 MPa, 2.44 MPa, 3.21 MPa, and 3.45 MPa, respectively. At this dosage, the reinforcing effect of PA in combination with cement was slightly inferior to that of pure cement and the 5% PA mixtures. When the PA dosage further increased to 15%, the UCS values of 0C + 15A, 5C + 15A, 10C + 15A, and 15C + 15A samples were 0.21 MPa, 0.74 MPa, 1.35 MPa, and 1.76 MPa, respectively. These results indicate a significant reduction in strength, implying that a 15% PA content produces a poor reinforcement effect. This may be due to the reduced cement content at high PA levels; insufficient Ca(OH)_2_ is available to react with the SiO_2_ and Al_2_O_3_ in PA, leaving excess PA unreacted and ineffective in forming a stable binding structure.

### 3.2. Unconfined Compressive Strength After Dry–Wet Cycling

Fluctuations in groundwater levels can expose soft clay foundations to alternating wetting and drying conditions, creating a dynamic environment that may damage the internal structure of the reinforced clay. Therefore, assessing the strength variation of reinforced soft clay under dry–wet cycles is essential for evaluating its long-term durability. The dry–wet cycling procedure adopted in this study follows the method described in [24]: specimens were first vacuum-saturated for 3 h and then oven-dried at 105 °C for 8 h for a cycle.

The data in Table 2 indicate that the strength of all specimens remained nearly unchanged after the first dry–wet cycle. However, as the number of cycles increased, the strength gradually declined, with a pronounced decrease observed after five cycles. For pure cement-reinforced soft clay, the strength losses after five cycles at cement contents of 15%, 20%, 25%, and 30% were 37.11%, 31.31%, 26.03%, and 24.17%, respectively. This trend suggests that a higher cement content enhances the material’s resistance to strength degradation under dry–wet cycling. The underlying mechanism is that greater cement content generates more hydration products, which effectively fill internal pores and increase the density of the clay matrix.

As shown in Table 3, the strength loss rates of specimens reinforced with 5% PA–cement (10C + 5A, 15C + 5A, 20C + 5A, and 25C + 5A) after five dry–wet cycles were 29.06%, 22.76%, 20.23%, and 19.55%, respectively. In contrast, the specimens reinforced with 10% PA–cement (5C + 10A, 10C + 10A, 15C + 10A, and 20C + 10A) exhibited much higher strength losses of 80.65%, 49.59%, 31.15%, and 30.43%, respectively (Table 4). These results clearly demonstrate that the soft clay reinforced with 5% PA–cement possesses superior resistance to dry–wet cycling, whereas the mixtures containing 10% PA–cement exhibit the weakest durability. The underlying mechanism of the above phenomenon can be attributed to the pozzolanic activity of PA when used in moderate amounts. When 5% PA is incorporated with cement, its reactive silica and alumina components effectively promote secondary hydration reactions. On one hand, this process consumes additional pore water within the soft clay matrix, thereby reducing the lubricating water film thickness between clay layers. On the other hand, it generates a greater quantity of hydration products, which fill internal pores and enhance the structural compactness of the material. Moreover, the ettringite and other hydration products formed during the secondary reaction are highly hydrophilic and may expel water molecules from the interlayer regions of clay minerals [30,31]. This reduces the thickness of interlayer water films, strengthens interparticle interactions, and ultimately improves both the mechanical strength and durability of the reinforced soft clay. In contrast, when the PA content reaches 10%, the excessive amount of ash cannot be fully consumed during cement hydration. Owing to its inherently porous structure and low intrinsic strength, the unreacted biomass ash increases the total porosity of the composite matrix, resulting in a deterioration of mechanical performance and dry–wet cycle resistance.

Based on the combined results of unconfined compressive strength and dry–wet cycle tests, the optimal reinforcement scheme is achieved by incorporating 5% PA with cement. This mixture not only provides the highest strength but also exhibits superior durability under dry–wet cycling conditions.

### 3.3. Solidification Mechanism

#### 3.3.1. Hydration Heat Analysis

**Figure 4:**
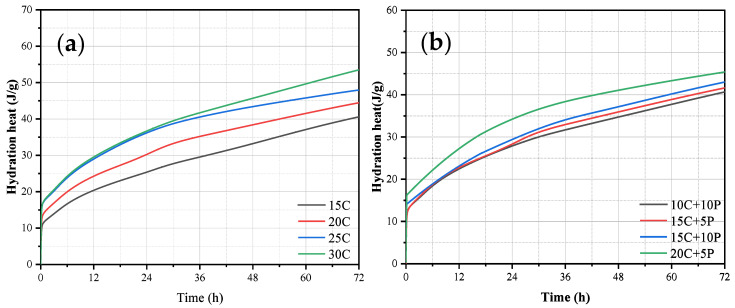
The hydration heat results of soft clay: (**a**) pure cement sample: (**b**) PA–cement sample.

Cement hydration is an exothermic and highly vigorous chemical process, and the heat of hydration released during this reaction is commonly used as an indicator of the intensity of early-stage reactions [32,33]. It can be seen that there is a relationship between the cumulative heat release of cement and the early strength of cement-reinforced soil. Figure 4a presents the hydration heat evolution of soft clay reinforced with pure cement at varying cement contents. It can be observed that the cumulative heat of hydration increases with cement content, indicating that a higher proportion of cement participates in the hydration process and consumes more water. This, in turn, promotes the continuous development of early strength in the cement-stabilized samples.

The hydration heat release characteristics of the PA–cement systems are shown in Figure 4b. A comparative analysis reveals that the total heat release of the PA–cement mixtures is lower than that of the pure cement systems at the same overall stabilizer dosage. Specifically, the 20% cement sample releases 43.81 J/g of heat within 72 h, whereas the 15C + 5A and 10C + 10A samples, each with a total stabilizer content of 20%, release only 41.34 J/g and 40.23 J/g, respectively. Similarly, the 25% cement sample exhibits a total heat release of 47.94 J/g, while the corresponding 20C + 5A and 15C + 10A mixtures release 45.24 J/g and 43.37 J/g, respectively. These results demonstrate that the incorporation of PA reduces the total heat of hydration. This phenomenon suggests that PA is largely inert during the early hydration stage and reacts minimally with water [18]. PA reacts with the Ca(OH)_2_ to generate more hydration products. However, in the early hydration stage, there is not a lot of Ca(OH)_2_ in the matrix, so PA cannot participate in the early hydration reaction. Consequently, its presence delays cement hydration and lowers the early strength of the reinforced soft clay. The trend of hydration heat evolution is therefore consistent with the observed 3-day strength behavior: PA does not actively participate in early hydration, and its incorporation diminishes early strength development; the higher the PA content, the more pronounced this adverse effect.

#### 3.3.2. XRD Analysis

**Figure 5:**
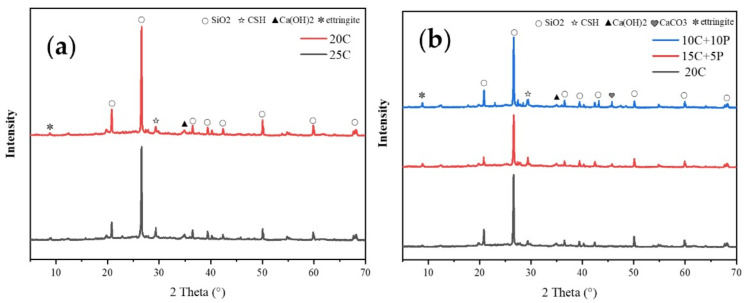
The XRD results of soft clay: (**a**) pure cement sample: (**b**) PA–cement sample.

XRD was employed to characterize the phase composition and relative content of hydration products. Figure 5 presents the XRD patterns of soft clay reinforced with different proportions of stabilizers at 28 days. Figure 5a shows the results for pure cement-reinforced soft clay. Prominent SiO_2_ diffraction peaks are observed, which can be attributed to the abundant silicon–oxygen tetrahedral structures in quartz and clay minerals present in the soft clay [34,35,36]. In addition, diffraction peaks corresponding to Ca(OH)_2_ and C-S-H were clearly identified. Notably, as the cement content increases, the peak intensities of Ca(OH)_2_ and C-S-H also increase, indicating a higher degree of cement hydration and the generation of more hydration products. In contrast, the intensity of the SiO_2_ peaks decreases slightly, which may be due to partial participation of SiO_2_ from the clay matrix in the hydration reactions.

Figure 5b shows the XRD patterns of PA–cement-reinforced soft clay. It is observed that increasing PA content leads to a decrease in the peak intensities of Ca(OH)_2_ and C-S-H. This is primarily due to the substitution effect of PA: a higher PA content reduces the relative proportion of cement, thereby limiting hydration reactions. Meanwhile, PA itself is rich in SiO_2_, and its excessive incorporation introduces an abundance of silica. However, the available calcium in the PA–cement system becomes insufficient in reacting with all the silica, resulting in an increase in unreacted SiO_2_ and enhanced diffraction peak intensity. Notably, CaCO_3_ and ettringite were also detected in the PA–cement system. PA has a significant CO_2_ capture capacity, which promotes the carbonation of Ca(OH)_2_ to form CaCO_3_ [17]. Furthermore, the 15% cement + 5% PA (15C + 5A) sample exhibits slightly lower SiO_2_ peak intensity compared with the 20% cement sample, whereas the peaks of C-S-H and ettringite are stronger. This indicates that the addition of an appropriate amount of PA can effectively promote secondary hydration, generating more C-S-H and ettringite. The enhancement of these hydration products is the key reason why the 15C + 5A sample exhibits higher strength than both the pure cement and the 10C + 10A samples.

#### 3.3.3. TG Analysis

**Figure 6:**
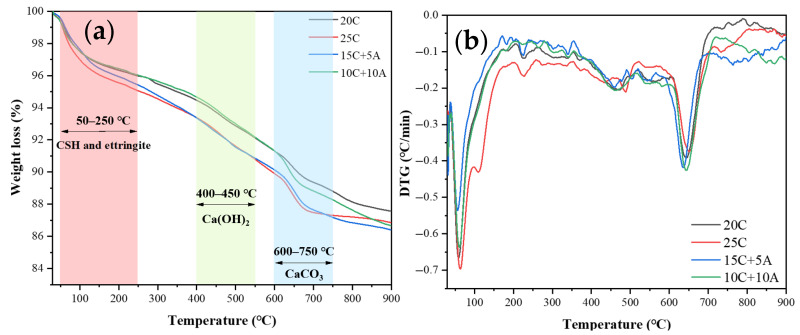
The thermogravimetric analysis results of soft clay: (**a**) weight loss curve: (**b**) DTG curve.

Hydration products such as ettringite, C-S-H, and Ca(OH)_2_ all contain crystal water, which volatilizes during calcination, leading to a reduction in sample mass. In addition, CaCO3 decomposes within a specific temperature range due to the release of CO_2_. By quantitatively analyzing the mass loss of samples over different temperature ranges, the relative content of the corresponding hydration products can be inferred. Based on references [29,37], 50–250 °C correspond to the dehydration of C-S-H and ettringite; 400–550 °C correspond to the dehydration of Ca(OH)_2_; and 600–750 °C correspond to the decomposition of CaCO_3_. The mass change curves as a function of calcination temperature are shown in Figure 6a. By applying a differential treatment to these curves, the mass loss rate (DTG) during calcination can also be obtained, as presented in Figure 6b. The detailed mass losses of each sample within the three characteristic temperature ranges are summarized in Table 5.

For pure cement samples, the mass loss in all three temperature intervals increases with cement content, indicating that a higher cement proportion generates a greater amount of hydration products. In the 5% PA–cement samples, the mass losses in the 50–250 °C and 400–550 °C ranges are higher than those of the 10% PA–cement samples, suggesting that the 5% PA system achieves a higher degree of hydration and forms more C-S-H, ettringite, and Ca(OH)_2_ in the matrix. Notably, the mass loss of the 5% PA–cement sample in the 50–250 °C range even exceeds that of the 25% pure cement sample, indicating that the incorporation of 5% PA promotes the formation of C-S-H and ettringite and enhances matrix hydration. Conversely, the 10% PA–cement samples exhibit significantly higher mass loss in the 600–750 °C range compared with other groups. This may be attributed to the strong CO_2_ adsorption capacity of PA [17]; an increased PA content facilitates carbonation, converting more Ca(OH)_2_ into CaCO_3_. Additionally, the mass losses of 10% PA–cement samples in the 50–250 °C and 400–550 °C ranges are lower than those of the pure cement samples, further confirming that excessive PA dilutes the cement content and inhibits hydration.

Overall, the formation of hydration products revealed by XRD and TG analysis aligns well with the observed unconfined compressive strength and dry–wet cycle performance. Specifically, the 5% PA–cement system effectively enhances cement hydration, produces a denser matrix, and significantly improves the mechanical properties of the reinforced soft clay. In contrast, 10% PA over-dilutes the cement, restricts hydration, and consequently weakens the strength of the reinforced clay.

#### 3.3.4. SEM Analysis

**Figure 7:**
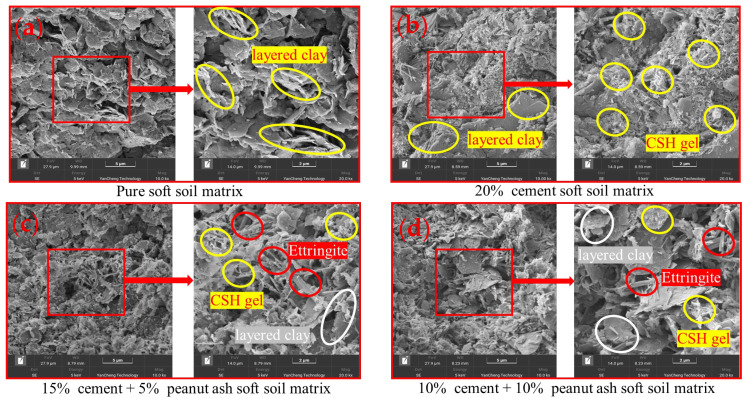
The morphology of soft clay: (**a**) soft clay: (**b**) 20% cement; (**c**) 10% cement + 5% peanut ash; (**d**) 10% cement + 10% peanut ash.

The quantity of hydration products influences the microstructure of clay, thereby affecting the density of the matrix. Figure 7a shows the microstructure of untreated soft clay without any stabilizer, where numerous layered structures are loosely stacked. These layered structures correspond to hydrophilic clay minerals such as montmorillonite and kaolinite [38,39,40], which strongly adsorb water molecules on their surfaces or within the interlayers, limiting water mobility.

Figure 7b illustrates soft clay reinforced with 20% cement. Partial C-S-H gel is formed in the matrix and is distributed at the interfaces of the clay layers, effectively filling pores and increasing structural compactness. In Figure 7c, the 15C + 5A-reinforced soft clay exhibits a greater amount of C-S-H, along with abundant needle-like ettringite. These products are interspersed on the surfaces of the clay sheets, resulting in a denser matrix structure.

The microstructure of the 10C + 10A-reinforced soft clay shown in Figure 7d is similar to that of the 15C + 5A sample, but the quantity of C-S-H and ettringite is relatively lower, and the pore-filling effect is limited. Notably, soft clay reinforced with PA–cement exhibits more ettringite compared with pure cement-reinforced samples. This observation further confirms that the pozzolanic components in PA participate in the secondary hydration of cement, promoting the formation of additional hydration products and enhancing the consolidation of the soft clay matrix.

## 4. Conclusions

This study investigated the reinforcement effect of peanut ash combined with cement on soft clay through unconfined compressive strength testing and dry–wet cycle experiments, and further analyzed the influence mechanism of peanut ash on the cement hydration process. The main conclusions are as follows:

(1) When cement is used alone as the stabilizer material, it exhibits a pronounced early-strengthening effect. The unconfined compressive strength of the stabilized soft clay increases with cement content, and the later strength of the specimen reinforced with 20% cement reaches 2.81 MPa. Therefore, a cement content of approximately 20% can be considered an appropriate range for the effective stabilization of soft clay using pure cement.
(2) When peanut ash is combined with cement for soft clay stabilization, its incorporation adversely affects the early strength development. However, beyond a curing age of 7 days, the inclusion of 5% peanut ash enhances both the strength and dry–wet cycle resistance of the reinforced soil. The strength of the specimen with 15% cement + 5% peanut ash reaches 3.12 MPa, which is 11.03% higher than that of the specimen reinforced with 20% pure cement, and comparable to the 3.15 MPa obtained with 25% cement. After five dry–wet cycles, the strength reduction of the 15% cement + 5% peanut ash sample is 22.76%, compared to 31.31% for the 20% cement sample, indicating superior durability. The mixture of 15% cement + 5% peanut ash is identified as the optimal reinforcement scheme.
(3) Results from hydration heat, XRD, TG, and SEM analyses demonstrate that peanut ash does not participate in the early hydration reaction, leading to a reduction in early hydration heat release. However, peanut ash can engage in secondary hydration reactions, promoting the formation of additional C-S-H gel and ettringite. These products effectively fill the inter-lamellar pores within the clay matrix, significantly enhancing the compactness and structural integrity of the stabilized soil. When the peanut ash content increases to 10%, the dilution effect on cement particles becomes dominant, reducing the quantity of hydration products and thereby exerting a detrimental influence on the mechanical properties of the solidified soft clay.

This study primarily validates the feasibility of using peanut ash in conjunction with cement to reinforce soft soils. It conducts an analysis of the strength and resistance to dry–wet cycling of the reinforced soil. However, it does not compare the enhancement effects of peanut shell ash against other agricultural biomass ashes, nor does it evaluate its carbon emission benefits. Moreover, it also does not consider the durability of the reinforced soil under various environmental conditions. Future research should be further refined to enhance the applicability boundary of peanut shell ash.

## Figures and Tables

**Table 1:** The ratio of cement to peanut ash.

Name	CaO	SiO_2_	Al_2_O_3_	MgO	SO_3_	Fe_2_O_3_	Others
Cement	64.13%	21.43%	4.67%	2.87%	2.43%	3.51%	0.96%
Peanut ash	4.89%	64.32%	16.34%	3.12%	1.06%	6.23%	4.04%

**Table 2:** The strength of pure cement-reinforced soft clay after the dry–wet cycle.

Cycle Time	15C	20C	25C	30C
0	1.94 MPa	2.81 MPa	3.15 MPa	3.31 MPa
1	1.82 MPa	2.66 MPa	2.94 MPa	3.24 MPa
3	1.63 MPa	2.34 MPa	2.72 MPa	2.93 MPa
5	1.22 MPa	1.93 MPa	2.33 MPa	2.51 MPa

Note: “C” represents cement.

**Table 3:** The strength of 5% peanut ash and cement-reinforced soft clay after the dry–wet cycle.

Cycle Time	10C + 5A	15C + 5A	20C + 5A	25C + 5A
0	2.03 MPa	3.12 MPa	3.41 MPa	3.64 MPa
1	1.92 MPa	2.98 MPa	3.37 MPa	3.55 MPa
3	1.81 MPa	2.71 MPa	3.13 MPa	3.25 MPa
5	1.44 MPa	2.41 MPa	2.72 MPa	2.93 MPa

Note: “C” represents cement and “A” represents peanut ash.

**Table 4:** The strength of 10% peanut ash and cement-reinforced soft clay after dry–wet cycle.

Cycle Time	5C + 10A	10C + 10A	15C + 10A	20C + 10A
0	0.62 MPa	2.44 MPa	3.21 MPa	3.45 MPa
1	0.54 MPa	2.02 MPa	3.02 MPa	3.25 MPa
3	0.31 MPa	1.74 MPa	2.73 MPa	2.84 MPa
5	0.12 MPa	1.23 MPa	2.21 MPa	2.40 MPa

Note: “C” represents cement and “A” represents peanut ash.

**Table 5:** The detailed mass loss of soft clays after calcination.

Cement Soft Clay				Cement and Peanut Ash Soft Clay			
	50–250 °C	400–550 °C	600–750 °C		50–250 °C	400–550 °C	600–750 °C
20C	3.558	2.544	2.491	15C + 5A	4.154	2.503	2.956
25C	4.083	2.569	2.596	10C + 10A	3.303	2.499	3.045

## Data Availability

The original contributions presented in this study are included in the article. Further inquiries can be directed towards the corresponding author.
